# Genotypic and ecological variability of zinc content in the grain of spring bread wheat varieties in the international nursery KASIB

**DOI:** 10.18699/VJ21.061

**Published:** 2021-09

**Authors:** V.P. Shamanin, P. Flis, T.V. Savin, S.S. Shepelev, O.G. Kuzmin, A.S. Chursin, I.V. Pototskaya, I.E. Likhenko, I.Yu. Kushnirenko, A.A. Kazak, V.A. Chudinov, T.V. Shelaeva, A.I. Morgounov

**Affiliations:** Omsk State Agrarian University named after P.A. Stolypin, Omsk, Russia; University of Nottingham, Nottingham, United Kingdom; Kazakh Research Institute of Agriculture and Plant Growing, Almalybak, Almaty region, Kazakhstan; Omsk State Agrarian University named after P.A. Stolypin, Omsk, Russia; Omsk State Agrarian University named after P.A. Stolypin, Omsk, Russia; Omsk State Agrarian University named after P.A. Stolypin, Omsk, Russia; Omsk State Agrarian University named after P.A. Stolypin, Omsk, Russia; Siberian Research Institute of Plant Production and Breeding – Branch of the Institute of Cytology and Genetics of the Siberian Branch of the Russian Academy of Sciences, Novosibirsk, Russia; Chelyabinsk Agricultural Research Institute, Chelyabinsk, Russia; Northern Trans-Ural State Agricultural University, Tyumen, Russia; Karabalyk Experimental Agricultural Research Station, Karabalyk, Kostanai region, Kazakhstan; Research and Production Center for Grain and Farming named after A.I. Baraev, Shortandy, Akmola region, Kazakhstan; Ministry of Environment, Water and Agriculture, Riyadh, Kingdom of Saudi Arabia

**Keywords:** variety, grain of wheat, zinc, protein, ecology, сорт, зерно пшеницы, цинк, белок, экология

## Abstract

Spring bread wheat is the staple crop in Western Siberia and Kazakhstan, a signif icant portion of which goes for export. Wheat breeding with a high level of zinc in wheat grain is the most cost-effective and environmentally friendly way to address zinc def iciency in the diet. The purpose of this work was to evaluate the contribution of the factors ‘location’ and ‘genotype’ in the variability of zinc content in wheat grain, and to identify the best varieties as sources of this trait for breeding. The research on screening zinc content in the wheat grain of 49 spring bread wheat varieties from the Kazakhstan- Siberia Spring Wheat Trial (KASIB) nursery was carried out at 4 sites in Russia (Chelyabinsk, Omsk, Tyumen, Novosibirsk) and 2 sites in Kazakhstan (Karabalyk and Shortandy) in 2017–2018. The content of zinc in wheat grain was evaluated at the Ionomic Facility of University of Nottingham in the framework of the EU project European Plant Phenotyping Network-2020. The analysis of variance showed that the main contribution into the general phenotypic variation of the studied trait, 38.7 %, was made by the factor ‘location’ due to different contents of zinc and moisture in the soil of trial sites; the effect of the factor ‘year’ was 13.5 %, and the effect of the factor ‘genotype’ was 8.0 %. The most favorable environmental conditions for accumulation of zinc in wheat grain were observed in the Omsk region. In Omsk, the average zinc content in all studied varieties was 50.4 mg/kg, with 63.7 mg/ kg in the best variety ‘OmGAU 100’. These values are higher than the target values of the international program Harvest Plus. ‘Novosibirskaya 16’ (49.4 mg/kg), ‘Silach’ (48.4 mg/kg), ‘Line 4-10-16’ (47.2 mg/ kg), ‘Element 22’ (46.3 mg/kg) and ‘Lutescens 248/01’ (46.0 mg/kg) were identif ied as being the best varieties. Signif icant possibilities for the production of wheat grain with high zinc content, which is in demand for the production of bread and pastry products with functional properties, were identif ied in the Western Siberian region.

## Abstract (ru)

Яровая мягкая пшеница является основной культурой в Западной Сибири и Казахстане, где зна- чительная доля производимого зерна идет на экспорт. Селекция пшеницы на повышенное содержание цинка в зерне – наиболее рентабельный и экологичный способ решения проблемы дефицита цинка в рационе пи- тания. Цель настоящей работы – установить вклад факторов «пункт» и «генотип» в изменчивость содержания цинка в зерне пшеницы и выделить лучшие сорта в качестве источников данного признака для селекции. Ис- следования по скринингу накопления цинка в зерне пшеницы 49 сортов яровой мягкой пшеницы из питом- ника КАСИБ-18 проведены в четырех пунктах России (Челябинск, Омск, Тюмень, Новосибирск) и двух пунктах Казахстана (Карабалык и Шортанды) в течение 2017–2018 гг. Содержание цинка в зерне определяли на фа- культете иономики Университета г. Ноттингем в рамках проекта EPPN-2020. Результаты дисперсионного ана- лиза показали, что основной вклад в общее фенотипическое варьирование признака вносил фактор «пункт» (38.7 %) вследствие разного содержания цинка в почве и влагообеспеченности в пунктах испытания; влияние факторов «год» и «генотип» составило 13.5 и 8.0 % соответственно. Наиболее благоприятные экологические условия для получения зерна пшеницы с повышенным содержанием цинка сложились в Омской области, где в среднем по всем сортам содержание цинка было равно 50.4 мг/кг, а у лучшего сорта ОмГАУ 100 – 63.7 мг/ кг. Эти показатели выше целевых значений международной программы Harvest Plus. Выделены лучшие сорта – Новосибирская 16 (49.4 мг/кг), Силач (48.4 мг/кг), Линия 4-10-16 (47.2 мг/кг), Элемент 22 (46.3 мг/кг) и Лютес- ценс 248/01 (46.0 мг/кг). В Западно-Сибирском регионе выявлены значительные потенциальные возможности производства зерна пшеницы с повышенным содержанием цинка, востребованного для получения хлеба и кондитерских продуктов с функциональными свойствами.

## Introduction

Wheat remains one of three crop commodities (along with maize and rice) contributing to global food security. Global wheat production has been increasing at a steady annual rate of 1–2 % to meet the growing population demand. According to FAO (http://www.fao.org/faostat/en), in Russian Federation, area under wheat has grown from 23.9 mln ha in 2014 to 26.5 mln ha in 2018 (+10.9 %), grain yield – from 2.50 to 2.72 t/ha (+8.8 %) and the total production – from 59.7 to 72.1 mln t (+20.7 %). The grain exports have increased more than two times and exceeded 35 mln t in 2019 and over 38.5 mln t in 2020. At present, wheat production in the world satisfies the demand and more attention shall be paid to wheat grain quality.

One of the pioneering works on this subject is European Union Project HEALTH GRAIN. The project was implemented in 2005–2010 and laid out the foundation for the studies for improving grain wheat nutritional value: protein content and composition, carbohydrates, vitamins, micronutrients and phytochemicals (Björck et al., 2012). Unfortunately, in Russia, the work on functional properties of wheat grain is limited to the study of purple wheat and its products in the Institute of Cytology and Genetics (Khlestkina et al., 2019; Gordeeva et al., 2020). The enhancement of functional properties and nutritional value of wheat grain products will have beneficial effect on human health and immune status, especially in connection with threats similar to coronavirus pandemic.

Wheat biofortification was started in mid-2000s by Harvest Plus consortium (https://www.harvestplus.org/what-we-do/ crops) and made tremendous progress. The grain zinc concentration of new biofortified wheat varieties increased by 40 % (+12 mg/kg) compared to commercial varieties (Velu et al., 2011; Singh R., Velu, 2017).

Recent results obtained from Harvest Plus and Harvest Zinc projects in China, India, Mexico, Pakistan, South Africa, and Turkey indicate positive effects of foliar-applied Zn (zinc) alone, and a micronutrient cocktail solution containing I (iodine), Zn, Se (selenium), and Fe (iron) that significantly improve grain accumulation of micronutrients, particularly in new biofortified wheat varieties. Grain-Zn was increased from 28.6 to 46.0 mg/kg with Zn-spray and 47.1 mg/kg with micronutrient cocktail spray (Zou et al., 2019).

Grain Zn contents of wheat varied among different countries from 25.10 mg/kg in Europe to 33.91 mg/kg in North America depending on: (1) the amount of Zn available in the soil; (2) genotypic characteristics of cultivated varieties; (3) cultivation types, environments, climates (Wang et al., 2020). Modern wheat varieties have limited grain Zn concentration: on the average – 14–42 mg/kg (Bouis, 1995; Morgunov et al., 2007; Velu et al., 2011; Guttieri et al., 2015). In this connection a large-scale screening of wheat genetic resources at the germplasm bank of the International Maize and Wheat Improvement Center (CIMMYT) was initiated to explore variation for Zn amongst the wheat wild relatives T. monococcum, T. dicoccoides, Ae. tauschii, T. boeticum, T. spelta, T. polonicum, landraces, and wheat hexaploid synthetics, which detected the most promising sources for development of varieties with high grain Zn concentration (Cakmak et al., 2004; Velu et al., 2014; Verma et al., 2016; Savin et al., 2018; Bhatta et al., 2019).

A field evaluation of a set of core-collection of landraces of CIMMYT screened under Zn-enriched soil conditions at Cd. Obregon (Mexico) showed that there was high variation for grain Zn concentration – from 40 to 96 mg/kg. T. dicoccoides introgression lines with bread wheat background showed up to 88 mg/kg grain Zn concentration. The first high zinc wheat variety Zincol 2016, having T. spelta in its pedigree, was released in Pakistan. Zn-enriched wheat varieties such as Zinc Shakti, WB02, and HPBW 01 were adapted by more than 500,000 farmers in India. These varieties were developed using synthetic hexaploid wheat with the genome of Ae. tauschii (Velu et al., 2019).

V. Govindan et al. (2018) reported a moderate level of broad-sense heritability for grain Zn concentration, and a significant Genotype × Environment interaction effect on this trait. The search and introgression of genes controlling high zinc content into the initial material for grain quality breeding through marker assisted selection has been conducted. One study identified QTLs associated with grain Zn concentration in wheat. These were located on chromosomes 2A, 5A, 7A (Peleg et al., 2009; Xu et al., 2012; Krishnappa et al., 2017). According to the research results of Y. Genc et al. (2009), the combination of four loci located on chromosomes 7А, 4B, 6B, and 3D increased the grain Zn by 23 %. The gene GPC-B1 (NAM-B1) was transferred to bread wheat genome from T. dicoccoides. Current tetraploid and hexaploid wheat varieties have non-active allele GPC-B1, except for some landraces and old varieties of T. dicoccum, T. durum, T. spelta, and T. aestivum (Mitrofanova, Khakimova, 2016). The active allele of this gene can be effective in improving high protein content, and remobilization of micronutrients from flag leaf to grains, which increases the concentration of Fe and Zn by 18 and 12 %, respectively (Uauy et al., 2006; Waters et al., 2009).

Grain Zn concentration is negatively correlated with yield in spring wheat varieties in several studies (Welch, Graham, 2002; Morgunov et al., 2007; Murphy et al., 2008). Some experiments, on the contrary, indicate that this correlation does not necessarily occur, and illuminate the possibility of combining high grain Zn with high grain yield and protein content in new varieties (Chen et al., 2017; Krishnappa et al., 2017; Abugalieva, Savin, 2018). The minerals’ bioavailability to humans, including Zn, depends on the phytic acid, which binds them. In this connection, the current wheat varieties should combine high yield with low phytic acid/Zn ratio (<5) (Qi et al., 2013; Liu et al., 2014).

Omsk State Agrarian University (Omsk SAU) coordinates Kazakhstan-Siberia network on spring wheat improvement (KASIB), which combines 20 breeding and scientific research institutions from Kazakhstan and Russia. In earlier studies, more than 150 genotypes were evaluated at 4–8 sites of KASIB network in Kazakhstan and Western Siberia in search for genetic resources of high zinc content. The relationship of Zn grain concentration with protein content and effects of Genotype × Environment interaction for this trait were studied (Morgоunov et al., 2006; Gomez-Becerra et al., 2007).

In 2016, Kazakh Research Institute of Farming and Crop Production won a grant of the project EPPN-2020 (European Plant Phenotyping Network) to conduct ionomics analysis of spring wheat grain from Kazakhstan and Russia using ionomics phenotyping platform at the University of Nottingham (UK). This platform couples high throughput elemental analysis based on automated data capture and their processing with bioinformatic methods (https://www.ionomicshub.org/ home/PiiMS). Ionomics analysis of 23 elements including heavy and rare metals, micronutrients in 49 spring bread wheat varieties at 6 sites in Kazakhstan and Russia was conducted in 2017–2018. This study identified that the variability of elemental analysis of grain wheat depends on the Genotype × Environment factors, and their interaction. The highest Zn and Fe concentrations in grain wheat were detected in Omsk oblast. Some varieties and breeding lines with high Zn and Fe content were identified (Abugalieva et al., 2020).

The objective of this research was to determine the contribution of the ‘Location’ and ‘Genotype’ factors on variability of wheat grain Zn content, and to select the best varieties as sources of this trait for breeding.

## Materials and methods

**Fig. 1.**
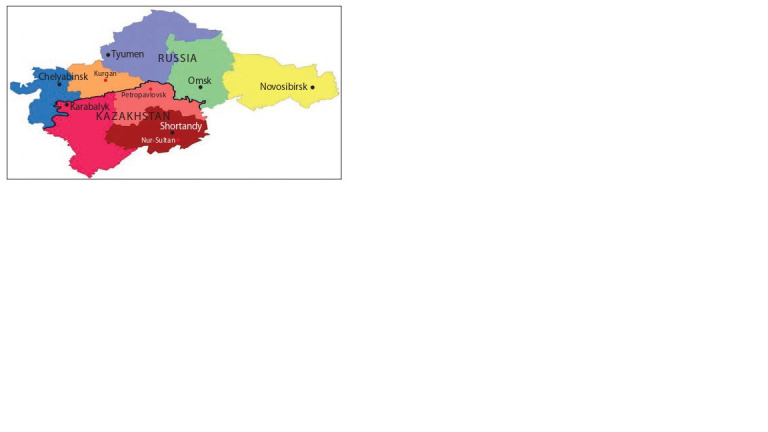
Map of experimental sites of nursery KASIB-18 in the regions of Russia and Kazakhstan in 2017 and 2018.

The study of 49 varieties of spring bread wheat of the nursery KASIB-18 (Kazakh-Siberian nursery of spring bread wheat) was carried out at four sites in Western Siberia, Southern Urals, and in two sites in Kazakhstan (Fig. 1). Geographic coordinates of Russian experimental sites are: Chelyabinsk Research Institute of Agriculture (Chelyabinsk) – 54°93′ N, 60°73′ E; Omsk SAU (Omsk) – 55°01′ N, 73°18′ E; Northern Trans-Ural SAU (Tyumen) – 57°09′ N, 65°25′ E; Siberian Research Institute of Plant Cultivation and Breeding (Novosibirsk) – 54°89′ N, 82°97′ E; Kazakhstan experimental sites: Karabalyk Experimental Agricultural Research Station (Karabalyk) – 53°51′ N, 62°06′ E; Research and Production Center for Grain and Farming (Shortandy) – 51°63′ N, 71°04′ E.

Variety trial of the nursery KASIB-18 was carried out in 2017–2018. The weather conditions differed significantly in geographic experimental sites (Table 1).

**Table 1.**
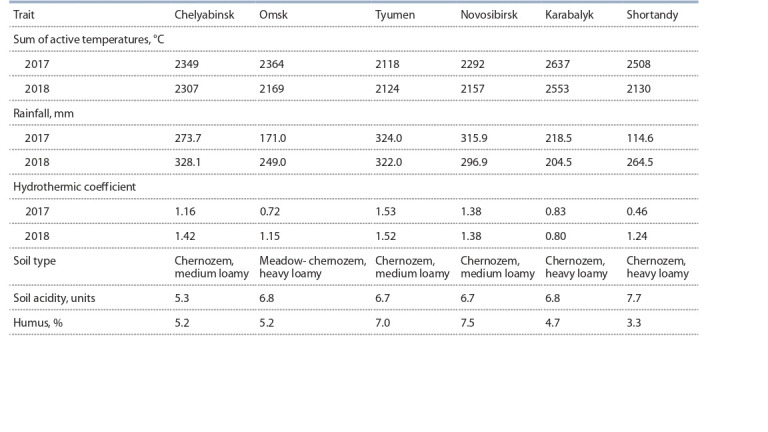
Characteristics of soil and weather conditions at experimental sights during the growing season (May–September), 2017–2018

At all experimental sites of the KASIB network, the sum of active temperatures above 10 °C in 2017–2018 was higher than the values necessary for normal growth and development of wheat plants: the lowest at Tyumen – 2118–2124 °C and the highest at Karabalyk – 2553–2637 °C. According to the hydrothermic coefficient (HTC), calculated by G.T. Selyaninov method (1958), the most favorable conditions for moisture availability were in the sites Tyumen and Novosibirsk (HTC = 1.38–1.53) in both years of research, as well as in Chelyabinsk in 2018 (HTC = 1.42), which had a positive effect on the formation of higher grain yield in these sites. In general, 2017 was characterized by drier conditions in Omsk (HTC = 0.72) and Shortandy (HTC = 0.46) compared to 2018 (HTC = 1.15 and 1.24, respectively). In Karabalyk, in both years, dry conditions were observed during the plant growing season (HTC = 0.80–0.83).

There are no significant differences in the soil morphological characters of the experimental sites, with the exception of higher humus content in Tyumen and Novosibirsk (7.0–7.5 %). Based on the literature sources, zinc content in the humus layer of meadow chernozem soils of the Omsk region is 20.1–69.4 mg/kg (Azarenko et al., 2019). According to the data from JSC “Kazakhstan Agrarian Expertise” branch (www. kazagrex.kz) in Akmola region, zinc content in low-humus soil of Shortandy is 3.3 mg/kg. The data of zinc content in the soil of the remaining sites are not available.

Sowing, selection assessments, and observations in the nursery were carried out in accordance with the Methodology of State Variety Trial of Agricultural Cultures (1989) and the program of the Kazakhstan-Siberia network on spring wheat improvement. The plot area was 3 m2 with a sowing rate of 500 seeds per 1 m2. The sowing date was May 20–30, the sowing depth – 4–5 cm. Field trials utilized a systematic complete block design with three replicates. The preceding crop was black fallow.

Grain samples from each experimental site were analyzed in the Kazakh Research Institute of Agriculture and Plant Growing (Almalybak), for protein content in grain and its fractions determined by Kjeldahl method (State standard No. 10846-91) using Infratec FOSS 1841 on the basis of previously created calibration equations. Zinc content in grain was determined at the Ionomics Faculty of the University of Nottingham. Zinc concentration was calculated in mg/kg of dry weight. Statistical data processing was reconstructed by variational, correlation, and ANOVA analysis using Microsoft Excel and Statistica application software packages.

## Results

Analysis of zinc accumulation in wheat grain of 49 varieties of KASIB-18 indicates significant differences in the grain zinc content, depending on the experimental site (Table 2).

**Table 2.**
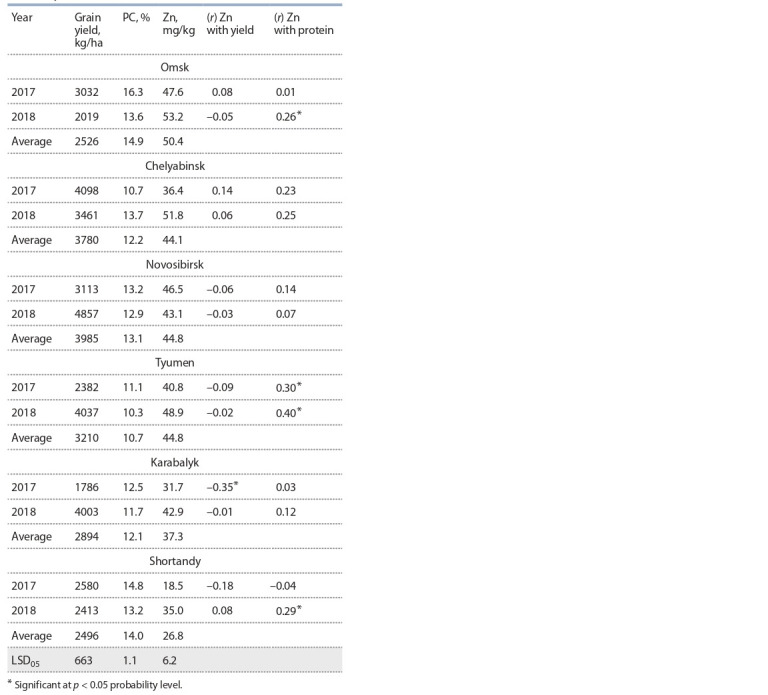
Grain yield, protein content (PC), Zn content, and coeff icients of correlation between (r) Zn content, yield and PC in the varieties from experimental sights of nursery KASIB-18 in 2017–2018

In Omsk, the yield was low, but the grain Zn content, on the contrary, was the highest (50.4 mg/kg). In Shortandy, the average grain yield was almost at the level of the varieties yield in Omsk, but the grain Zn content was 1.9 times lower, which indicates a significant influence of soil and climatic properties of the region on the accumulation of this microelement in wheat grain. The highest grain Zn content was found in Omsk and in other sites of Russia – Tyumen, Novosibirsk, Chelyabinsk (44.1–44.8 mg/kg), and significantly less in Kazakhstan – Karabalyk and Shortandy (37.3 and 26.8 mg/kg, respectively).

No correlation was found between grain Zn content and yield. One site was an exception – under dry conditions of 2017, an average negative relationship was observed in Karabalyk (r = –0.35) with the lowest yield (1786 kg/ha) compared to the rest experimental sight.

On average, for two years of research, the highest grain yield was in Novosibirsk – 3985 kg/ha, in Tyumen, and Chelyabinsk – 3210 and 3780 kg/ha, respectively. The yield obtained in Karabalyk, Omsk, and Shortandy was less than 3000 kg/ha. Significant differences in the grain average protein content of different experimental sites were found. The highest protein content on average for two years of research was observed in Omsk (14.9 %), in Shortandy (14.0 %), in Novosibirsk (13.1 %), in Chelyabinsk (12.2 %), in Karabalyk (12.1 %), and the lowest – in Tyumen (10.7 %). In Tyumen, in 2017–2018, an average positive correlation was observed between grain Zn and protein content – 0.3 and 0.4, respectively.

**Fig. 2.**
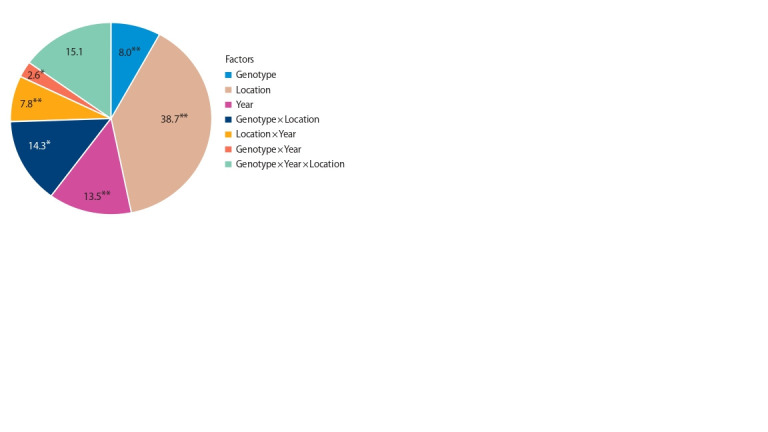
Contribution of various factors for Zn grain content of wheat varieties, %. Signif icant at * p < 0.05 and ** p < 0.001 probability level.

On the basis of the experiment results of 49 varieties for two years in 6 ecological sites, a three-factor ANOVA analysis was carried out, and the contribution of the main factors to variability of wheat grain Zn was determined (Fig. 2). The main contribution to the variability of the studied trait was made by the ‘Location’ factor – 38.7 %. ANOVA analysis revealed significant influence of the following factors: ‘Year’ – 13.5 %, ‘Genotype – 8.0 %, ‘Genotype × Location’ – 14.3 %, and ‘Location × Year’ – 7.8 %. The combined effect of three factors interaction was significant – 15.1 %.

**Fig. 3.**
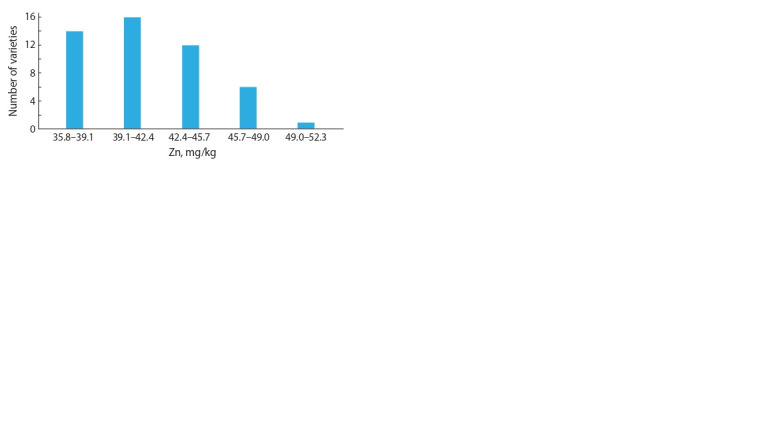
Number of varieties by intervals of Zn grain content from experimental sites, on average of 2017–2018.

Figure 3 presents the limits of average indicators of grain Zn content of the studied varieties for two years of research. The maximum grain Zn content was observed in one variety – 49.4 mg/kg, in six varieties Zn content varied from 45.7 to 49 mg/kg, in 16 varieties – from 39.1 to 42.4 mg/kg, in 14 varieties – from 35.8 to 39.1 mg/kg, in 12 varieties – from 42.4 to 45.7mg/kg. The varieties with a high grain Zn content identified by the experiment results on average for all sites for two years, and the variability limits of the trait, depending on year and site, are presented in Table 3. The differences between the presented varieties on the average zinc content in grain correspond to error of the experiment.

**Table 3.**
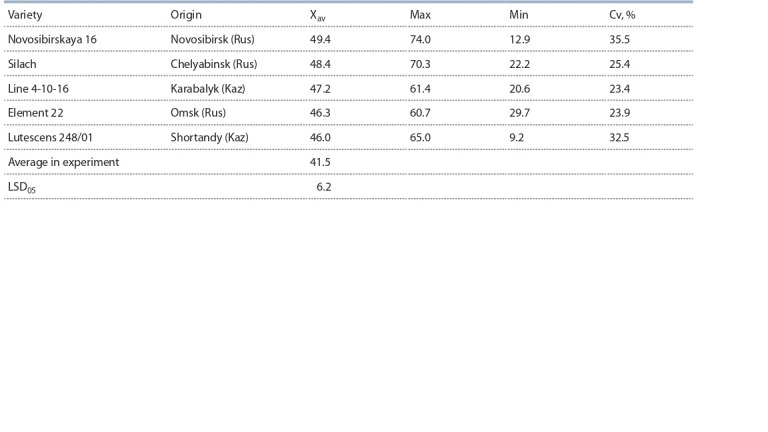
Varieties with the highest Zn grain content (mg/kg) from experimental sites, on average of 2017–2018

Variety Novosibirskaya 16 on average had Zn content of 49.4 mg/kg, but this indicator was not stable, the trait value varied from 12.9 to 74.0 mg/kg, the variation coefficient was 35.5 %. Varieties Silach, Line 4-10-16, and Element 22 were more stable than Novosibirskaya 16. On average, the grain Zn content of studied varieties was 41.5 mg/kg.

**Fig. 4.**
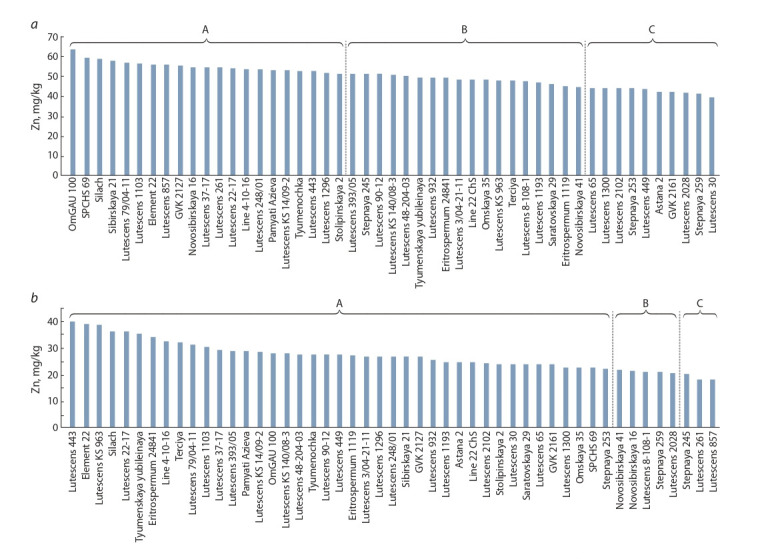
Ranging of varieties of nursery KASIB-18 depending on Zn grain content, on average for 2017–2018: a, Omsk; b, Shortandy. Different letters indicate homogeneous groups.

Figure 4, a presents the distribution of varieties in Omsk by the grain Zn content. The highest content of this microelement for all varieties was noticed in this site in all years of research. According to the ranging of varieties by the grain Zn content three groups were distinguished: 21 varieties (51.6– 63.7 mg/kg) were assigned to the first, 18 varieties (44.7– 51.3 mg/kg) were assigned to the second, and 10 varieties with a relatively low microelement content (39.4–44.4mg/kg) were assigned to the third. The significant differences on Zn content were noticed for varieties of the first and third groups as the contribution of the genotypic factor to expression of studied trait was 8 %. The highest zinc content of 49 varieties was observed in variety OmGAU100 – 63.7mg/kg on average for two years, and the lowest – in variety Lutescens 30 – 39.4 mg/kg.

In Shortandy, there were no significant differences among varieties on grain Zn content: the first group included 42 varieties (21.9–39.7 mg/kg), the second and third – seven varieties (18.2–21.5 mg/kg). Variety Lutescens 443 had the highest grain Zn content – 39.7 mg/kg, which is almost at the level of the lowest indicator of the trait in Omsk (see Fig. 4, b). Variety Lutescens 857 was characterized by low grain Zn content (only 18.2 mg/kg) in Shortandy.

Thus, varieties OmGAU 100 and Lutescens 443 selected in two experimental sites should also be included in the hybridization program for the improvement of wheat grain Zn content.

## Discussion

According to the World Health Organization, two billions of people worldwide are at risk of suffering from Zn deficiency (WHO, 2017). The regions where zinc deficiency is most common are Southeast Asia, southern Africa, and other developing countries. The main factors leading to Zn deficiency should first of all be attributed to insufficient consumption of Zn with food in the regions due to low content of food with low Zn content in soil, drought climate and lack of moisture (http://cgon.rospotrebnadzor.ru/content/62/2683).

The health benefits of wheat grain and products for consumers are a strategic priority worldwide (Saleh et al., 2019). Biofortified wheat with higher Zn concentration has proven its positive effect on human health in India and Pakistan (Listman et al., 2019). Russia is one of the world’s leading wheat exporters, exporting mainly to the Middle East and Africa, where there is a significant Zn shortage in the products of poor population eating mainly bread. Breeding wheat with enhanced levels of grain Zn provides a cost-effective, sustainable solution to the malnutrition problems in the developing world (Bouis et al., 2011).

Corresponding to our research, wheat grain grown in the experimental sites of Western Siberian and Ural regions has an increased Zn content on average from 44.1 to 50.4 mg/kg (see Table 2). In the sites Shortandy and Karabalyk, the grain Zn content was on average 26.8–37.3 mg/kg for two years of research. These data are consistent with the publication by J. Wang et al. (2020), according to which the Zn content in grain grown in Kazakhstan is on average 28.4 mg/kg. This is probably explained by dry climate in Kazakhstan. The plants need a mass flow of soil solution and ions for effective mineral nutrition, which depends primarily on the presence of moisture in the soil (Singh B. et al., 2005).

The ANOVA analysis revealed the influence of genotypic factor, soil, climatic, and weather conditions on the grain Zn accumulation in studied wheat varieties. The main contribution to the trait phenotypic variation was made by the ‘Location’ factor due to different Zn content in the soil and moisture availability in the experimental sites – 38.7 %. The influence of the ‘Year’ factor was 13.5 % and the ‘Genotype’ factor – 8.0 %.

In most sites of research, 2018 was more favorable on the available moisture in the soil compared to dry 2017. In 2018, grain Zn content varied from 35.0 mg/kg (Shortandy) to 53.2 mg/kg (Omsk). In 2017, under drought conditions, lower grain Zn concentration was noticed in Karabalyk (31.7 mg/kg), 18.5 mg/kg – in Shortandy, 36.4 mg/kg – in Chelyabinsk, and 47.6 mg/kg – in Omsk (see Table 2).

According to the two years research results of the nursery KASIB-18 in all experimental sites, sources of high Zn content were identified: Novosibirskaya 16 (49.4 mg/kg), Silach (48.4 mg/kg), Line 4-10-16 (47.2 mg/kg), Element 22 (46.3 mg/kg), and Lutescens 248/01 (46.0 mg/kg). These indicators are higher than the target ones of the international Harvest Plus program. This program allowed to increase the grain Zn content in the wheat varieties cultivated in India and Pakistan from 25 mg/kg on average to 37 mg/kg, approximately by 30–40 % (Singh R., Velu, 2017).

There was high variability of the trait in selected varieties (Cv = 23.4–35.5 %), which indicates a strong influence of environmental conditions on this trait (see Table 3). The study of the same varieties set under contrasting environmental conditions revealed differences among varieties and lines in grain Zn accumulation. Varieties OmGAU 100 (63.7 mg/kg) and Lutescens 443 (39.4 mg/kg) were characterized by the maximum grain Zn accumulation in Omsk and Shortandy, while varieties Lutescens 30 (39.4 mg/kg) and Lutescens 857 (18.2 mg/kg) were characterized by the minimum, respectively (see Fig. 4, a, b).

A.V. Volkov (2015) reported the positive effect of zinc fertilizers on yield, protein content, gluten, technological, and bread making qualities in agrochemical experiments on the spring wheat variety Zlata. In our experiments, the average positive correlation between the grain Zn and protein content (0.3 and 0.4) was revealed in Tyumen for both years of research, as protein content was low (on average 10.7 %). In the other five sites, the correlation was not found. There is no correlation between yield and grain Zn content (see Table 2). Probably, the grain Zn content is not an indicator of the necessity to use Zn-fertilizers. This issue requires additional research under the conditions of a specific region and different soil types.

Varieties Novosibirskaya 16, Silach, Element 22, OmGAU 100 included in the State Register of Breeding Achievements and cultivated on large areas under conditions of Western Siberian region, are able to form Zn content in grain exceeding 60–70 mg/kg (see Table 3, Fig. 4, a). The analysis of wheat grain from farmer’s crops in the Omsk region also revealed a high grain Zn content. It is feasible to use it both in the domestic market for the production of bread and bakery products with functional properties, and for the export of wheat grain (Abugalieva et al., 2020). Our research confirms the high potential of Omsk, Novosibirsk, Tyumen, and Chelyabinsk regions for the production of grain with a high Zn content. It is advisable to use the grain of the best varieties on the Zn content to form separate batches for the production of “healthy” bread and for export. These are unused reserves that will contribute to the improvement of human health, especially for people with low incomes, as well as increase the export potential of the country.

## Conclusion

According to the research results, the most favorable soil and climatic conditions to form grain wheat with a high Zn content were in the Omsk region. On average, during two years of research of the nursery KASIB-18 in Omsk, the grain Zn content of the studied varieties was 50.4 mg/kg, which is more than in other Russian experimental sites – Tyumen, Novosibirsk, and Chelyabinsk (44.1–44.8 mg/kg). In Kazakhstan, the average grain Zn content of wheat varieties was 37.3 mg/kg in Karabalyk and 26.8 mg/kg in Shortandy. Significant differences on the grain Zn content among the varieties indicate the possibility of breeding improvement of wheat on this trait. On average, for all sites, for two years of research, the highest grain Zn content was revealed in varieties Novosibirskaya 16 (49.4 mg/kg), Silach (48.4), Line 4-10-16 (47.2), Element 22 (46.3), and Lutescens 248/01 (46.0 mg/kg). In the conditions of Omsk, variety OmGAU 100 (63.7 mg/kg), and in Shortandy, under less favorable conditions, variety Lutescens 443 (39.7 mg/kg) were distinguished. These varieties should be included in the hybridization program for the improvement of wheat grain Zn content.

The main contribution (38.7 %) to the variability of the grain Zn content was made by soil and climate conditions of the region (the ‘Location’ factor). Significant influences of the ‘Genotype’ factor – 8.0 %, and the ‘Year’ factor – 13.5% were revealed. There was no correlation between grain Zn content and yield, the correlation between grain Zn and protein content was revealed in Tyumen (–0.3 and –0.4). The potential opportunities for production of wheat grain with a high Zn content, which will be in demand for production of bread and pastry with functional properties, were identified in Western Siberian region.

## Conflict of interest

The authors declare no conflict of interest.
